# Quorum Sensing Activity of *Mesorhizobium* sp. F7 Isolated from Potable Water

**DOI:** 10.1155/2014/874764

**Published:** 2014-08-06

**Authors:** Pei-Ling Yong, Kok-Gan Chan

**Affiliations:** Division of Genetics and Molecular Biology, Institute of Biological Sciences, Faculty of Science, University of Malaya, 50603 Kuala Lumpur, Malaysia

## Abstract

We isolated a bacterial isolate (F7) from potable water. The strain was identified as *Mesorhizobium* sp. by 16S rDNA gene phylogenetic analysis and screened for *N*-acyl homoserine lactone (AHL) production by an AHL biosensor. The AHL profile of the isolate was further analyzed using high resolution triple quadrupole liquid chromatography mass spectrometry (LC/MS) which confirmed the production of multiple AHLs, namely, *N*-3-oxo-octanoyl-L-homoserine lactone (3-oxo-C8-HSL) and *N*-3-oxo-decanoyl-L-homoserine lactone (3-oxo-C10-HSL). These findings will open the perspective to study the function of these AHLs in plant-microbe interactions.

## 1. Introduction

Bacterial cell-cell communication, also termed quorum sensing (QS), occurs when the cell density has reached a threshold level that could regulate gene expressions via signalling molecules [1, 2]. Acyl-homoserine lactones (AHLs) molecules which are responsible for QS activity are synthesized by AHL synthase (LuxI homologue) whereby AHL will bind to its cognate receptor (LuxR homologue) to form a AHL-LuxR complex which in turn modulates a battery of QS-mediated gene expression [3, 4]. Upon sensing the AHLs, the Proteobacteria population works in synchrony to generate a significant impact on the bacterial physiological activities including bioluminescence, antibiotic production, plasmid conjugal transfer, and synthesis of exoenzyme virulence factors in plant and animal pathogens [5, 6]. Structurally, AHL consists of a conserved homoserine lactone moiety and the acyl chain length that ranges from 4 to 18 carbons [7].

Bacteria are commonly known to perform intra- or interspecies communication with the help of AHL. For example, a study on the interaction between* Serratia liquefaciens* MG1 and* Solanum lycopersicum* (tomato) gave first indications that AHL molecules of rhizosphere bacteria stimulate plant defense responses [8].

Rhizobia which include the genera* Rhizobium*,* Sinorhizobium*,* Mesorhizobium*,* Azorhizobium*,* and Bradyrhizobium* exhibit nitrogen fixing properties in root nodules of legumes which can only be achieved when bacteria carry large self-transmissible genetic elements, either plasmids or integrating conjugative elements that include symbiotic genes [9]. Plant growth is often limited by the availability of nitrogen, and, hence, plant-rhizobia interactions are particularly important for sustainable and environment-friendly crop production without the use of nitrogen fertilizers [10].

A key mechanism in the symbiotic process is the involvement of QS [11]. Signaling and communication between the host and rhizobia are required for successful symbiotic interactions [12]. The various functions regulated by AHLs of* Rhizobium* and* Sinorhizobium* range from exopolysaccharide production [13], rhizosphere-related gene expression [14], and the conjugal transfer of pSym plasmids [15]. QS is also an important mechanism in other plant growth-promoting rhizobacteria [16].

AHLs have been identified in various rhizobial strains (Table 1). For example, a marine* Mesorhizobium* sp. was isolated that produced novel long-chain* N*-acyl-L-homoserine lactones, namely, 5-*cis*-3-oxo-C12-HSL and 5-*cis*-C12-HSL. It was shown that both these compounds were able to restore protease and pyoverdine production of an AHL-deficient* Pseudomonas aeruginosa* PAO1* lasI rhlI* double mutant, suggesting the AHLs could be used for intergenus signaling [26]. In other previous studies, when C4-HSL, C6-HSL, 3-oxo-C6-HSL, and 3-oxo-C8-HSL are applied to the plant, these AHLs promoted the growth of* Arabidopsis* [27–29] whereas 3-oxo-C10-HSL induced the formation of adventitious roots in mung beans [30]. On the other hand, 3-oxo-C14-HSL and 3-OH-C14-HSL induced resistance in* Arabidopsis* and barley plants towards biotrophic and hemibiotrophic pathogens [31].

In another study on* Rhizobium* sp. strain NGR234, it was found that the production of 3-oxo-C8-HSL led to activation of the transcriptional regulator TraR which significantly decreased the growth rate of NGR234 [15]. Moreover, it was shown that the regulatory gene TraI of* Rhizobium etli* CFN42 controls synthesis of 3-oxo-C8-HSL and conjugative plasmid transfer [17].

Here, we have identified the AHLs of* Mesorhizobium* sp. F7 which was isolated from potable water. We showed that this strain produces two AHLs, 3-oxo-C8-HSL and 3-oxo-C10-HSL.

## 2. Experimental Section

### 2.1. Sample Collection and Processing

A water sample was collected from domestic filtered water with nonwoven fabric and powdered activated carbon features installed in Petaling Jaya, Selangor (Malaysia). The tap was left open to flow for a few minutes before the sample was collected in a sterile plastic tube. The water sample was processed within an hour of sample collection. An aliquot of the water sample (100 *μ*L) was plated on Difco Reasoner's 2A agar (0.5 g/L proteose; 0.5 g/L casamino acids; 0.5 g/L yeast extract; 0.5 g/L dextrose; 0.5 g/L soluble starch; 0.3 g/L dipotassium phosphate; 0.05 g/L magnesium sulfate; 0.3 g/L sodium pyruvate) and incubated at 37°C under aerobic growth condition for 3 days. The observable different morphologies of the bacteria colonies were isolated and screened for AHL production using cross-streaking with* Chromobacterium violaceum* CV026 [32].

### 2.2. Isolation and Characterization of Isolate F7

The bacterial isolate F7 was found to exhibit QS properties among all isolates through an AHL biosensor screen (*Chromobacterium violaceum* CV026). The genomic DNA was extracted using a MasterPureTM DNA Purification Kit (EPICENTRE Inc., Madison, WI, USA). The isolate F7 was later characterized by analyzing its 16S rRNA gene. The 16S rRNA was PCR amplified using 27F forward primer (5′-AGAGTTTGATCMTGGCTCAG-3′), 515F forward primer (5′-GTGCCAGCMGCCGCGGTAA-3′), and 1525R reverse primer (5′-AAGGAGGTGWTCCARCC-3′) using a PCR mix (Promega Kit, Madison, WI, USA). The PCR amplification that was carried out consists of an initial denaturation at 94°C for 3 min, followed by 30 repeated cycles at 94°C for 30 s of denaturing, 60°C for 30 s of annealing, and 72°C for 1 min 30 s of extension, and a final extension of 72°C for 7 min. Product sequence alignment was done using GenBank Blastn database and phylogenetic analysis was done using molecular evolutionary genetic analysis (MEGA) version 5.2 [33].

### 2.3. AHL Extraction

A single pure colony of isolate F7 was cultured overnight in R2 broth [34] buffered to pH 6.5, with 3-(*N*-morpholino) propanesulfonic acid (MOPS, 50 mM, pH 6.5) at 37°C with shaking (220 rpm). The supernatant was extracted twice with 100 mL of acidified (0.1% v/v glacial acetic acid) ethyl acetate [35]. The AHL extract was dried and stored at −20°C prior to further analysis.

### 2.4. Measurement of Bioluminescence

Preliminary screening of AHLs production by isolate F7 was done using an AHL biosensor (*Escherichia coli* [pSB401]).* E*.* coli* [pSB401] harbors* lux* from the pSB401 plasmid which will produce bioluminescence activity when exogenous short chain AHLs are supplied [36]. Cell density bioluminescence measurements were done using an Infinite M200 luminometer-spectrophotometer (Tecan, Männedorf, Switzerland). To every well of a 96-well optical bottom microtitre plate, an aliquot of 200 *μ*L diluted (1 : 100)* E. coli* [pSB401] overnight culture in LB broth supplemented with tetracycline (20 *μ*g/mL) and 1 *μ*L extracted AHL were added [37]. Acetonitrile and synthetic 3-oxo-C6-HSL (250 pg/*μ*L) were used as the negative and positive standards, respectively. Results were indicated as relative light units/optical density (RLU/OD_495 nm_) against incubation time (hour) [38].

### 2.5. Identification of AHL by High Resolution Tandem Liquid Chromatography Quadrupole Mass Spectrometry (LC-MS/MS)

LC-MS/MS was performed using Agilent 6490 Triple Quadrupole LC/MS system (Agilent Technologies Inc., Santa Clara, CA, USA) and all experimental conditions were performed essentially as reported previously. The settings for the Agilent Mass Hunter software for MS spectra analysis were applied as described [39, 40].

## 3. Results and Discussion

### 3.1. Identification of a Bacterial Isolate from Potable Water

**Figure 1:**
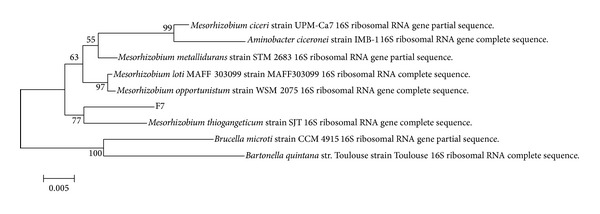
Phylogenetic tree of isolate F7.

Isolate F7 was identified by analyzing its 16S rRNA gene nucleotide sequence [41–43]. A phylogenetic tree was constructed using Mega 5.2 software to align it with other rRNA sequences obtained from GenBank. A total of 988 unambiguously aligned nucleotides were analysed using the Neighbor-Joining method. The percentage of replicate trees in which the associated taxa clustered together in the bootstrap test is shown next to the branches [42]. The bar represents evolutionary distance as 0.002 change per nucleotide. Based on the phylogenetic tree obtained from the 16S rRNA sequencing (Figure 1), F7 was identified as* Mesorhizobium* sp.

### 3.2. Production of AHL by* Mesorhizobium* sp. F7

**Figure 2:**
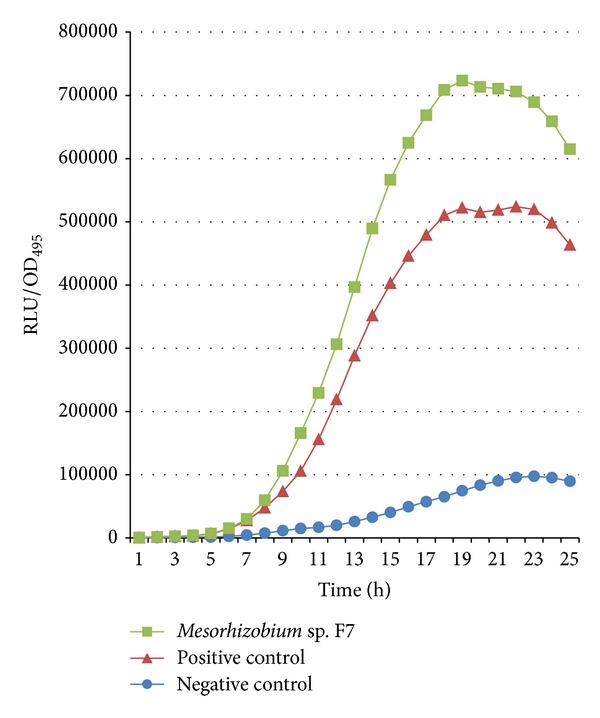
Detection of short chain AHLs production by* Mesorhizobium* sp.

Isolate F7 was screened for its production of short chain AHL molecules by using luminometer-spectrophotometer, where the activation of bioluminescence of the biosensor* E. coli* [pSB401] was observed (Figure 2). Bioluminescence measurement was done for 24 h, 37°C. Cells were grown in the presence of AHL extracted from culture supernatant of* Mesorhizobium* sp. and synthetic 3-oxo-C6-HSL and acetonitrile were used as positive and negative controls, respectively.

Data are presented as means of ± SEM values of triplicate experiments.

**Figure 3:**
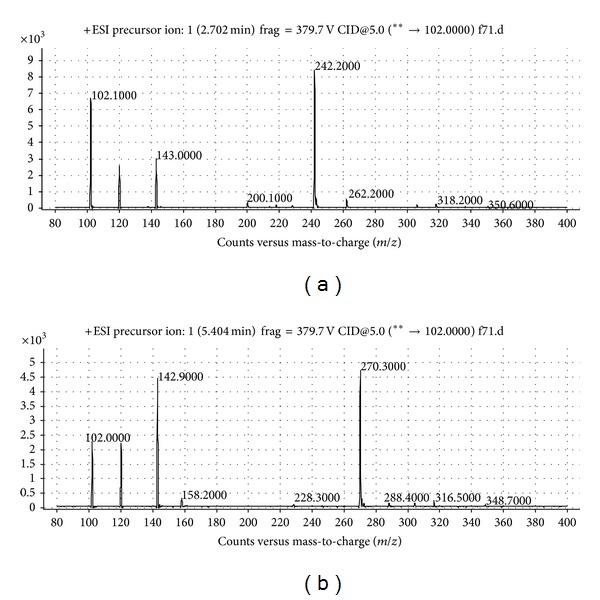
Mass spectrometry analysis of 3-oxo-C8-HSL: *m*/*z* value 242.000; retention time: 2.702 min; abundance: 8394.42 and abundance %: 100, and (b) 3-oxo-C10-HSL: *m*/*z* value: 270.200; retention time: 5.369 min; abundance: 3689.5 and abundance %: 100.

To identify the AHLs, triple quadrupole LC/MS analysis was used. The MS results of the material from culture supernatants of* Mesorhizobium* sp. F7 are presented in Figure 3. The data provide evidence for the presence of both short and long chain AHL molecules, namely, 3-oxo-C8-HSL (*m*/*z* 242.0000) and 3-oxo-C10-HSL (*m*/*z* 270.0000).

It was shown in other reports that the biological activity of AHLs applied to plants and the plant response is dependent on the length of lipid chains of AHLs. For instance, 3-oxo-C10-HSL was shown to be capable of inducing adventitious roots in mung bean [30] whereas 3-oxo-C8-HSL was active on* Arabidopsis* seedlings where it affected expression of 53 proteins related to plant primary metabolism, energy status, cytoskeleton, and defense [44]. Hence, we speculate that the AHLs produced by* Mesorhizobium* sp. F7 possess similar functions. Future experiments are required to test whether rhizobial strains producing specific AHLs can be used as biocontrol bacteria to stimulate plant defense reactions against plant pathogens.

## Figures and Tables

**Table 1:** AHL production in rhizobia.

AHL	Representative organisms
3-oxo-C8-HSL	*Rhizobium* sp. strain NGR234, *Rhizobium leguminosarum, Sinorhizobium meliloti,* and* Rhizobium etli* CFN42 [15, 17–21]
3-OH-C8-HSL	*R. leguminosarum, S. meliloti, *and *R. etli* CFN42 [17, 19, 22]
C8-HSL	*R. leguminosarum* and* S. meliloti* [14, 17, 21–24]
3-OH-C14-HSL	*R. leguminosarum* [25]
C16-HSL	*S. meliloti* [19]
